# Supplementary material for: Funcionality assessed by the core set of the international classification of functionality and health for rheumatoid arthritis: A cohort study
Source: PLoS One. 2023 Nov 8;18(11):e0286342. doi: 10.1371/journal.pone.0286342 (PMC10631651; doi:10.1371/journal.pone.0286342)
Supplement: Attachment — Submitted filename: Response to Reviewers.docx [file pone.0286342.s001.docx]

PONE-D-22-32670

Funcionality Assessed by the Core Set of the International Classification of Functionality and Health for Rheumatoid Arthritis: A cohort study.

Dear editor and reviewers,

Thank you for reading our manuscript, and for the careful and constructive reviews. In this letter we attempted to respond to all the comments and state the changes performed on the manuscript. We believe we have addressed the suggestions recommended by the reviewers and we are confident that the new version of the manuscript is greatly improved.

A revised version of the manuscript, with our changes highlighted in red (track changes), is attached, as well as an unmarked version of our revised paper without tracked changes. The responses to each point raised by the reviewers are detailed below. We look forward to hearing from you regarding our submission. And we would be glad to respond to any further questions and comments that you may have.

1. Please ensure that your manuscript meets PLOS ONE's style requirements, including those for file naming. The PLOS ONE style templates can be found at <https://journals.plos.org/plosone/s/file?id=wjVg/PLOSOne_formatting_sample_main_body.pdf> <https://journals.plos.org/plosone/s/file?id=ba62/PLOSOne_formatting_sample_title_authors_affiliations.pdf>

REPLY: The formatting of titles, subtitles and tables were changed to follow the journal's rules.

2. Thank you for stating the following financial disclosure:

"https://www.gov.br/capes/pt-br

https://www.fipe.org.br/pt-br/ensino/extensao-curta-duracao/

This study was funded by the Research Incentive Foundation (FIPE) of the Hospital de Clínicas de Porto Alegre and the Coordination for the Improvement of Higher Education Personnel (CAPES).
FIPE was used to cover general research expenses and a CAPES grant was given to study author Juliana Portes. Funders did not participate in any stage of the study."

Please state what role the funders took in the study. If the funders had no role, please state: "The funders had no role in study design, data collection and analysis, decision to publish, or preparation of the manuscript."
If this statement is not correct you must amend it as needed.
Please include this amended Role of Funder statement in your cover letter; we will change the online submission form on your behalf.

3. Thank you for stating the following in the cover letter Section of your manuscript:

"This study was funded by Research Incentive Foundation (FIPE) of the Hospital de Clínicas de Porto Alegre and Coordination for the Improvement of Higher Education Persons (CAPES)."

We note that you have provided funding information that is not currently declared in your Funding Statement. However, funding information should not appear in the Acknowledgments section or other areas of your manuscript. We will only publish funding information present in the Funding Statement section of the online submission form.
Please remove any funding-related text from the manuscript and let us know how you would like to update your Funding Statement. Currently, your Funding Statement reads as follows:

"https://www.gov.br/capes/pt-br
https://www.fipe.org.br/pt-br/ensino/extensao-curta-duracao/

This study was funded by the Research Incentive Foundation (FIPE) of the Hospital de Clínicas de Porto Alegre and the Coordination for the Improvement of Higher Education Personnel (CAPES).
FIPE was used to cover general research expenses and a CAPES grant was given to study author Juliana Portes. Funders did not participate in any stage of the study."

Please include your amended statements within your cover letter; we will change the online submission form on your behalf.

REPLY: All mentions of funders were removed from the article, however we had to include the phrase "We thank the Fundo de Incentivo à Pesquisa e Eventos of Hospital de Clínicas de Porto Alegre for their support." in the acknowledgments so that the value of the publication is subsidized and the role of the funders was stated in the cover letter.

4. We note that you have indicated that data from this study are available upon request. PLOS only allows data to be available upon request if there are legal or ethical restrictions on sharing data publicly. For more information on unacceptable data access restrictions, please see http://journals.plos.org/plosone/s/data-availability#loc-unacceptable-data-access-restrictions.

In your revised cover letter, please address the following prompts:

a) If there are ethical or legal restrictions on sharing a de-identified data set, please explain them in detail (e.g., data contain potentially sensitive information, data are owned by a third-party organization, etc.) and who has imposed them (e.g., an ethics committee). Please also provide contact information for a data access committee, ethics committee, or other institutional body to which data requests may be sent.

b) If there are no restrictions, please upload the minimal anonymized data set necessary to replicate your study findings as either Supporting Information files or to a stable, public repository and provide us with the relevant URLs, DOIs, or accession numbers. For a list of acceptable repositories, please see http://journals.plos.org/plosone/s/data-availability#loc-recommended-repositories.

We will update your Data Availability statement on your behalf to reflect the information you provide.

REPLY: The requested clarification was given in the cover letter.

5. Please review your reference list to ensure that it is complete and correct. If you have cited papers that have been retracted, please include the rationale for doing so in the manuscript text, or remove these references and replace them with relevant current references. Any changes to the reference list should be mentioned in the rebuttal letter that accompanies your revised manuscript. If you need to cite a retracted article, indicate the article’s retracted status in the References list and also include a citation and full reference for the retraction notice.

REPLY: The reference "Smolen, J. S. Rheumatoid arthritis. Lancet. 2016 May 3." was removed from the manuscript because it was refuted and despite being retracted, we understand that errors can influence the final outcome of the article.

The reference "Cruz-Jentoft, A.J.; Bahat, G.; Bauer, J.; Boirie, Y.; Bruyère, O.; Cederholm, T.; Cooper, C.; Landi, F.; Rolland, Y.; Sayer , A. A.; et al. Sarcopenia: revised European consensus on definition and diagnosis. Age Ageing 2019, 48 (1), 16-31." had a specific error in the table in Table 3, which shows the recommended cut-off points for ASM/height2 in women. The cut-off point was given as <6.0 kg/m2, but the correct value is <5.5 kg/m2. Which has been corrected in the online version: DOI: 10.1093/ageing/afy169. Erratum for: 10.1093/ageing/afy169. This error does not change any outcome of the article, remaining an important and reliable reference, so we chose to continue using it.

Some citations were removed from the references section because they were not being cited in the article but had not been removed from the references, they are:

1. McInnes, I. B.; Schett, G. The pathogenesis of rheumatoid arthritis. N Engl J Med 2011, 365 (23), 2205-2219. DOI: 10.1056/NEJMra1004965.
2. Eustáquio, C. V. F. d. S. AVALIAÇÃO DA LIMITAÇÃO AOS EXERCÍCIOS E SUA RELAÇÃO COM A FUNCIONALIDADE DE PACIENTES COM DPOC Revista CIF Brasil.: 2020.
3. Uhlig, T.; Lillemo, S.; Moe, R. H.; Stamm, T.; Cieza, A.; Boonen, A.; Mowinckel, P.; Kvien, T. K.; Stucki, G. Reliability of the ICF Core Set for rheumatoid arthritis. Ann Rheum Dis 2007, 66 (8), 1078-1084. DOI: 10.1136/ard.2006.058693.
4. Oude Voshaar, M. A.; Ten Klooster, P. M.; Glas, C. A.; Vonkeman, H. E.; Taal, E.; Krishnan, E.; Bernelot Moens, H. J.; Boers, M.; Terwee, C. B.; van Riel, P. L.; et al Validity and measurement precision of the PROMIS physical function item bank and a content validity-driven 20-item short form in rheumatoid arthritis compared with traditional measures. Rheumatology (Oxford) 2015, 54 (12), 2221-2229. DOI: 10.1093/rheumatology/kev265.
5. Chung, K. C.; Burns, P. B.; Reichert, H. A.; Fox, D. A.; Burke, F. D.; Wilgis, E. F.; Regan, M.; Kim, H. M. Properties of the International Classification for Functioning, Disability and Health in assessing hand outcomes in patients with rheumatoid arthritis. J Rehabil Med 2011, 43 (4), 292-298. DOI: 10.2340/16501977-0671. Strand, V.; Rentz, A. M.; Cifaldi, M. A.; Chen, N.; Roy, S.; Revicki, D. Health- related quality of life outcomes of adalimumab for patients with early rheumatoid arthritis: results from a randomized multicenter study. J Rheumatol 2012, 39 (1), 63-72. DOI: 10.3899/jrheum.101161.

5. Review Comments to the Author

Please use the space provided to explain your answers to the questions above. You may also include additional comments for the author, including concerns about dual publication, research ethics, or publication ethics. (Please upload your review as an attachment if it exceeds 20,000 characters)

Reviewer #1: This is an interesting informative manuscript on RA functionality assessment.

1. Typographical errors throughout the manuscript should be corrected, for example, in the Abstract- under "Objective" .....to evaluate "function" and not the functioning; under the" Results" -......over 12 months. The and not" the" . On page 4, " sence' should read; ' sense'

3. The same font should be used throughout the manuscript.

4. The word: " light" throughout the manuscript and in the Tables should be replaced by: " mild".

REPLY: All typographical errors have been corrected and all text is in the same font. All words light have been replaced by mild

2. The conclusions in the Abstract and at the end of the article should be rewritten to be illustrative of the results.
5. The Discussion section is somewhat weak-authors need to elaborate on their findings; they need to explain their results more comprehensively and defend their findings.

REPLY: We have modified our conclusion and amended our discussion to follow the reviewer's guidance.
